# Supplementary material for: Crystallographic Ensembles Reveal the Structural Basis of Binding Entropy in SARS-CoV2 Macrodomain
Source: bioRxiv. 2025 Dec 23:2025.11.25.690589. Originally published 2025 Nov 26. Preprint. [Version 2] doi: 10.1101/2025.11.25.690589 (PMC12699308; doi:10.1101/2025.11.25.690589)
Supplement: Supplement 3 [file NIHPP2025.11.25.690589v2-supplement-3.pdf]

bioRxiv preprint doi: https://doi.org/10.1101/2025.11.25.690589; this version posted December 23, 2025. The copyright holder for this preprint (which was not certified by peer review) is the author/funder, who has granted bioRxiv a license to display the preprint in perpetuity. It is made available under aCC-BY 4.0 International license.

Supplementary Figures

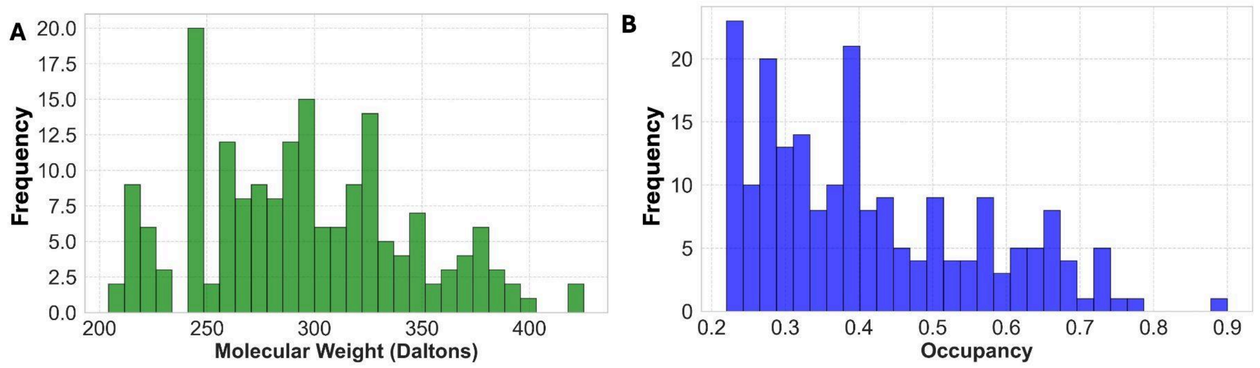

Supplementary Figure 1. A. Distribution of molecular weight (range: 204.23-425.25). **B.** Distribution of ligand occupancy (22%-90%).

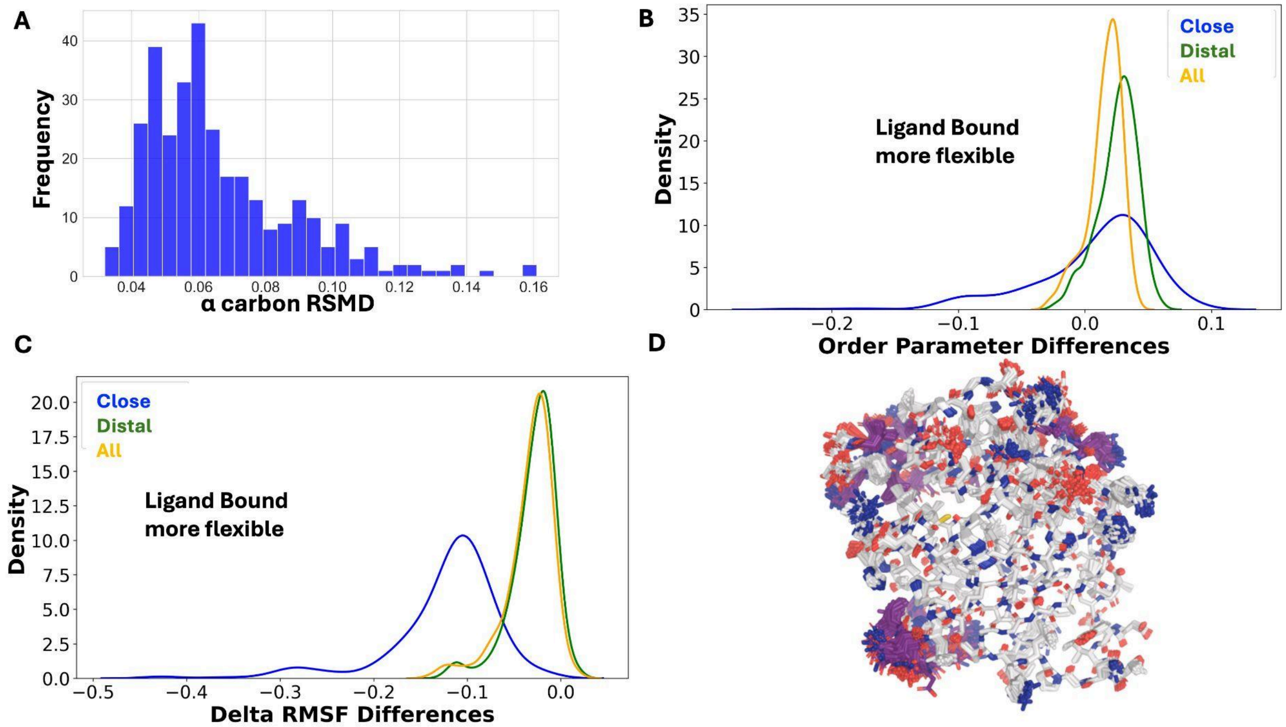

bioRxiv preprint doi: https://doi.org/10.1101/2025.11.25.690589; this version posted December 23, 2025. The copyright holder for this preprint (which was not certified by peer review) is the author/funder, who has granted bioRxiv a license to display the preprint in perpetuity. It is made available under aCC-BY 4.0 International license.

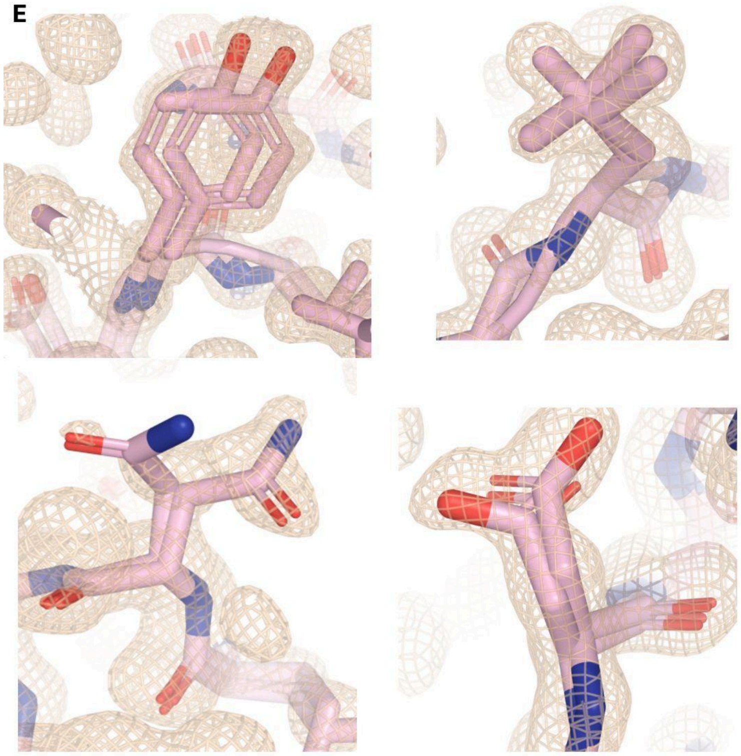

Supplementary Figure 2. A. Distribution of the alpha carbon RMSD values between apo and ligand-bound structures of Mac1. While most structures exhibit minimal backbone deviation, the distribution shows a long tail, indicating a subset of structures with larger backbone conformational differences. **B.** The average delta order parameter across all ligand-bound structures separated by location. Binding site residues are defined as those within 4 Angstroms of any ligand heavy ligand and distal residues as those more than 10 Angstroms from any ligand heavy atom **C.** The average delta RMSF across all ligand-bound structures separated by location. **D.** Example of ensemble refinement output with flexible regions colored purple. **E.** Examples of multiconformer side chains identified by qFit and used to model the anharmonic heterogeneity present.

bioRxiv preprint doi: https://doi.org/10.1101/2025.11.25.690589; this version posted December 23, 2025. The copyright holder for this preprint (which was not certified by peer review) is the author/funder, who has granted bioRxiv a license to display the preprint in perpetuity. It is made available under aCC-BY 4.0 International license.

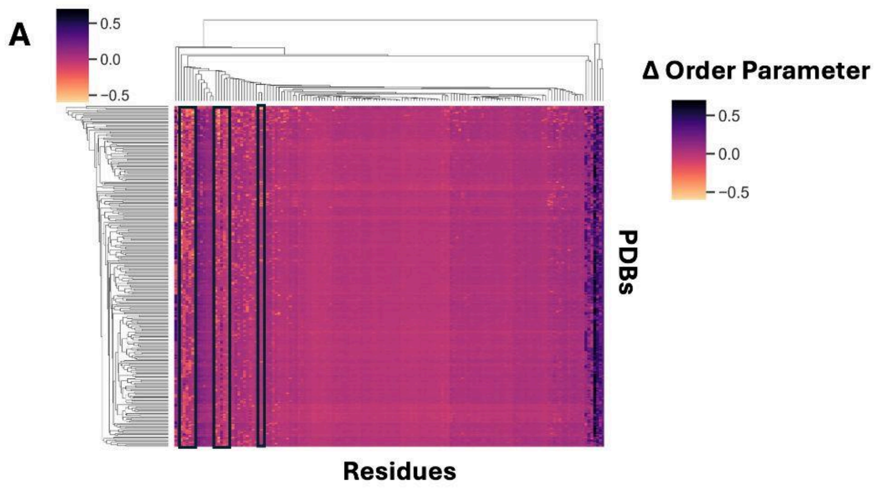

Supplementary Figure 3. A. Heatmap of residues that gain or lose conformational heterogeneity upon ligand binding across all macrodomain structures. Dark purple indicates an increase in conformational heterogeneity upon binding.

bioRxiv preprint doi: https://doi.org/10.1101/2025.11.25.690589; this version posted December 23, 2025. The copyright holder for this preprint (which was not certified by peer review) is the author/funder, who has granted bioRxiv a license to display the preprint in perpetuity. It is made available under aCC-BY 4.0 International license.

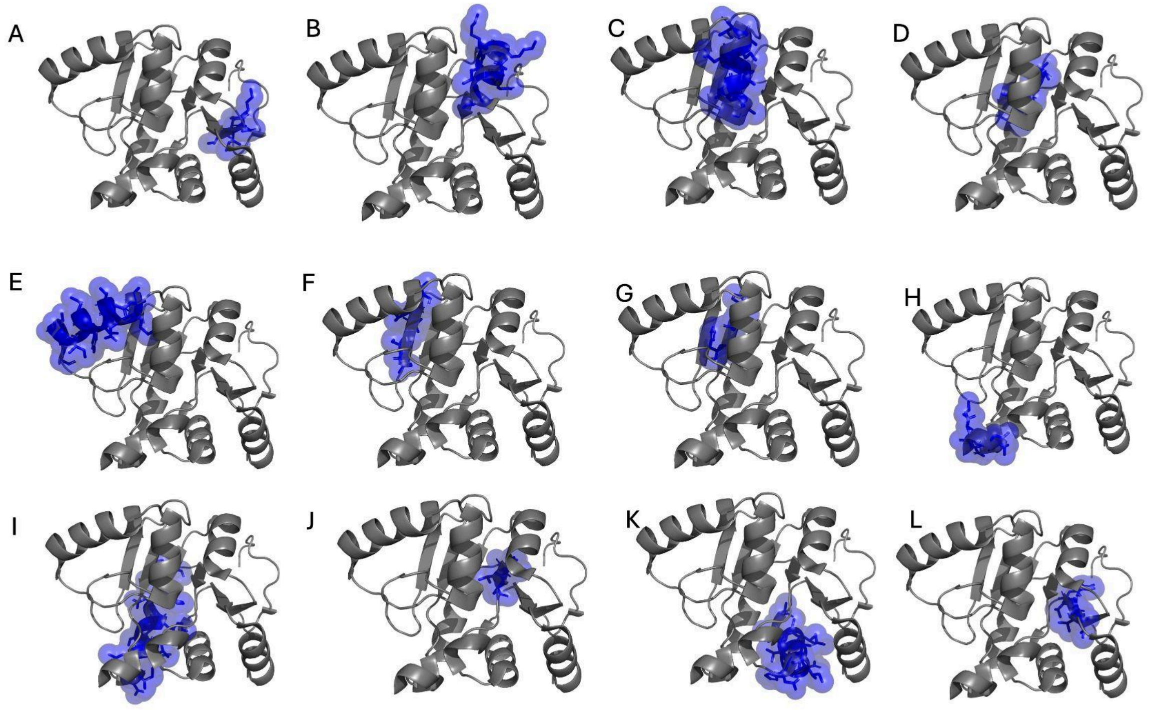

Supplementary Figure 4. Mac1 Locations **A.** beta sheet 1, **B.** alpha helix 1, **C.** alpha helix 2, **D.** beta sheet 2, **E.** alpha helix 3, **F.** beta sheet 3, **G.** beta sheet 4, **H.** loop 100-105, **I.** alpha helix 4, **J.** beta sheet 5, **K.** alpha helix 5, **L.** beta sheet 6

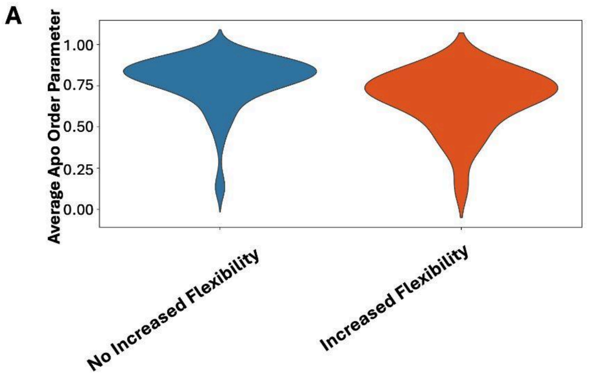

Supplementary Figure 5. A. Average apo crystallographic order parameter between residues identified has having repeatable increase in flexibility upon ligand binding v. those without repeatable increase in flexibility.

bioRxiv preprint doi: https://doi.org/10.1101/2025.11.25.690589; this version posted December 23, 2025. The copyright holder for this preprint (which was not certified by peer review) is the author/funder, who has granted bioRxiv a license to display the preprint in perpetuity. It is made available under aCC-BY 4.0 International license.

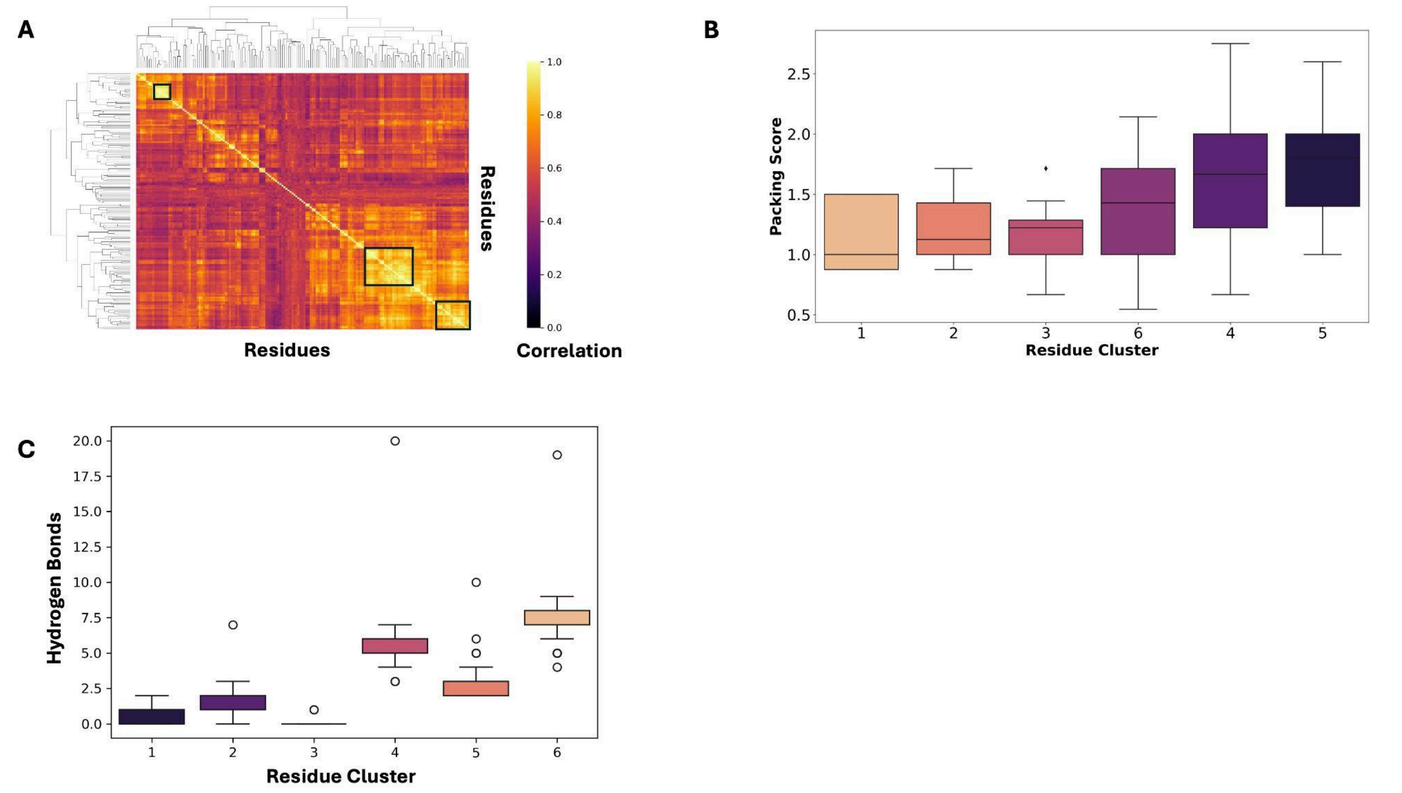

Supplementary Figure 6. A. The correlation matrix of residue flexibility, where positive correlations (yellow) indicate that residues tend to fluctuate together. **B.** Distribution of packing per residue cluster. Residue clusters with overall increased flexibility tend to be less well packed (clusters 1,2,3). In contrast, residues clusters with high co-correlation tend to be well packed (clusters 4,5,6). **C.** Distribution of hydrogen bonds within cluster residues (normalized by number of residues).

bioRxiv preprint doi: https://doi.org/10.1101/2025.11.25.690589; this version posted December 23, 2025. The copyright holder for this preprint (which was not certified by peer review) is the author/funder, who has granted bioRxiv a license to display the preprint in perpetuity. It is made available under aCC-BY 4.0 International license.

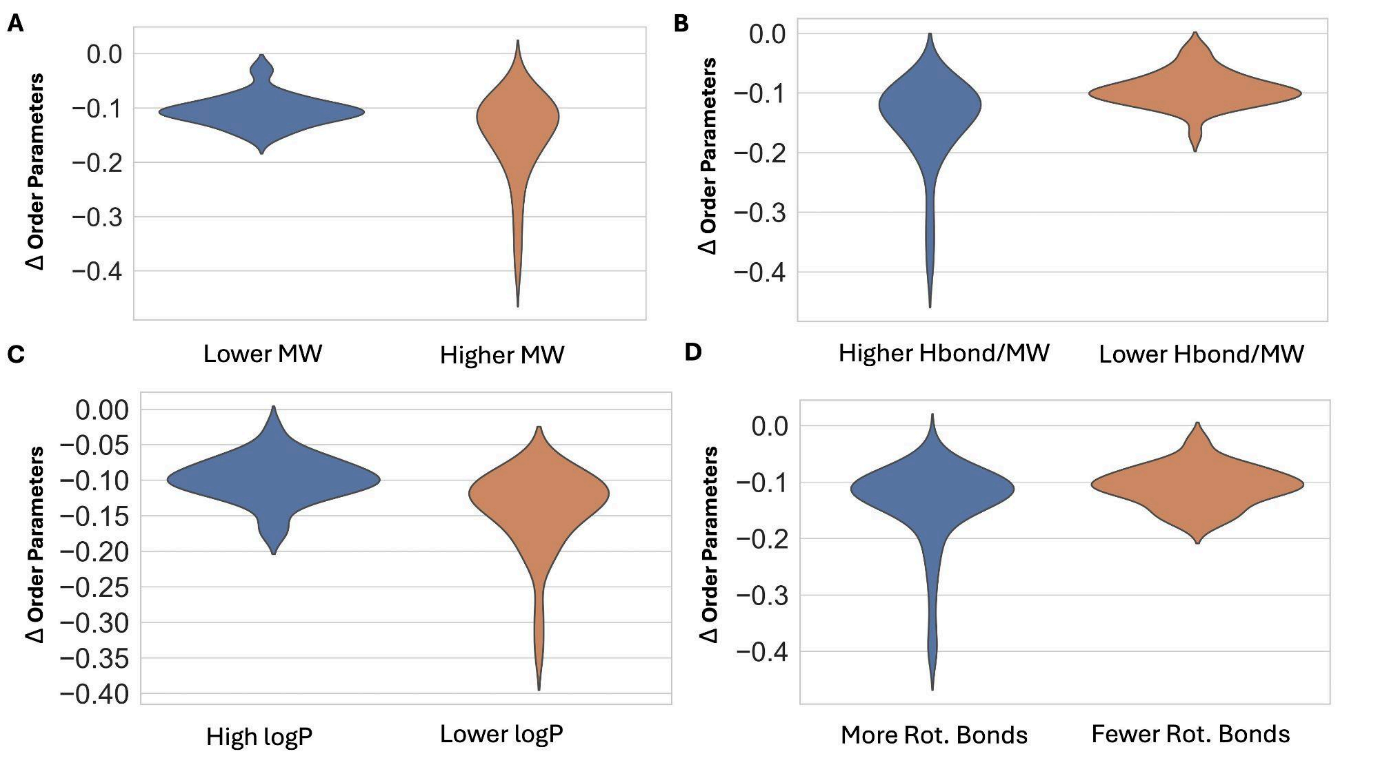

Supplementary Figure 7. Ligand properties correlation with conformational heterogeneity within the binding site. **A.** Higher molecular weight is more flexible (Higher Molecular Weight: ≥343.63 Da; ΔOP -0.11, Lower Molecular Weight: ≤ 270.38; ΔOP -0.11; p=0.01; Mann Whitney U test), **B.** More hydrogen bonds/molecular weight is less flexible (More Hbond/MW ≥0.04; ΔOP -0.09, Less Hbond/MW: ≤0.3; ΔOP -0.12; p=3.2e-05), **C.** High logP is more flexible (Higher logP ≥0.27; ΔOP -0.09, Lower logP ≤-1.17; ΔOP -0.09; p=9.5e-05) **D.** Higher number of rotatable bonds is more flexible (More rotatable bonds ≥5; ΔOP -0.11, Fewer rotatable bonds ≤3; ΔOP -0.11; p=0.03).

bioRxiv preprint doi: https://doi.org/10.1101/2025.11.25.690589; this version posted December 23, 2025. The copyright holder for this preprint (which was not certified by peer review) is the author/funder, who has granted bioRxiv a license to display the preprint in perpetuity. It is made available under aCC-BY 4.0 International license.

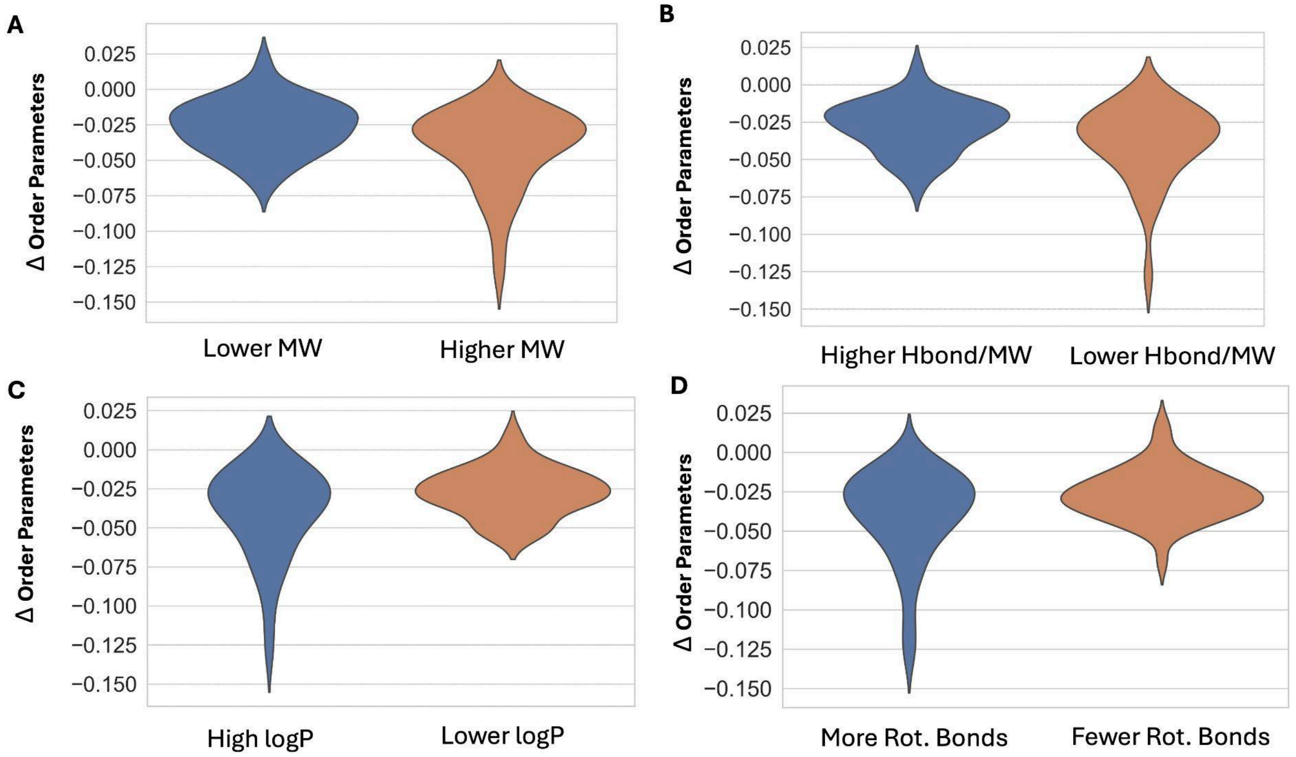

Supplementary Figure 8. Ligand properties correlation with conformational heterogeneity across the entire protein. **A.** Higher molecular weight is more flexible (Higher molecular weight ≥343.63 Da; ΔOP -0.03, Lower molecular weight ≤ 270.38; ΔOP -0.02; p=0.03; Mann Whitney U test), **B.** More hydrogen bonds/molecular weight is less flexible (More Hbond/MW ≥0.04; ΔOP -0.03, Fewer Hbond/MW≤0.3; ΔOP -0.03; p=3.2e-05), **C.** High logP is more flexible (Higher logP: ≥0.27; ΔOP -0.03, Lower logP: ≤-1.17; ΔOP -0.03; p=0.07) **D.** Higher number of rotatable bonds is more flexible (More rotatable bonds: ≥5; ΔOP -0.03, Fewer rotatable bonds: ≤3; ΔOP -0.02; p=0.07).

bioRxiv preprint doi: https://doi.org/10.1101/2025.11.25.690589; this version posted December 23, 2025. The copyright holder for this preprint (which was not certified by peer review) is the author/funder, who has granted bioRxiv a license to display the preprint in perpetuity. It is made available under aCC-BY 4.0 International license.

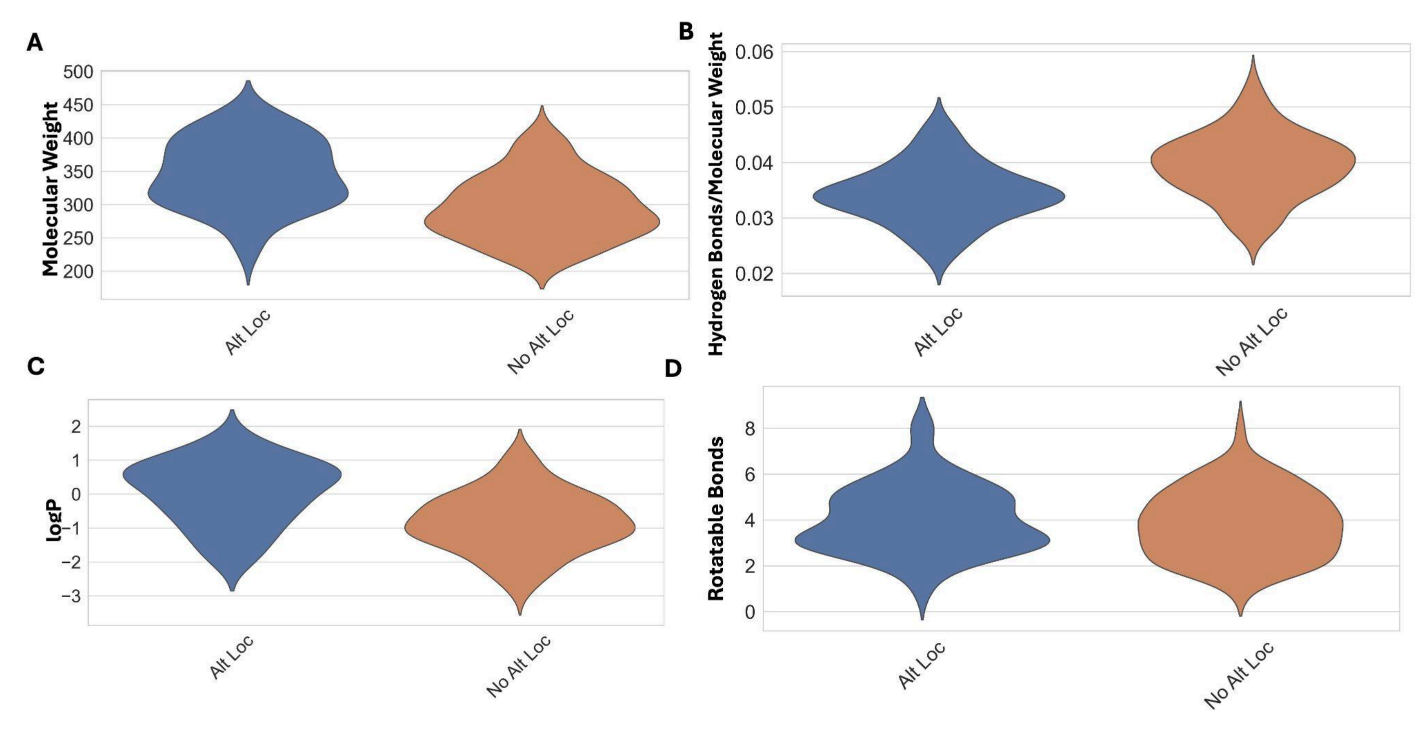

Supplementary Figure 9. Ligand property association with alternative conformers **A.** Molecular weight (Altloc median=343.9, non-altloc median= 286.91; p=4.39e-08), **B.** Hydrogen Bonds per molecular weight (Altloc median=0.034, non-altloc median= 0.04; p=5.90e-07), **C.** LogP (Altloc median=0.24, non-altloc median=-0.85; p=3.31e-07), **D.** Rotatable bond (Altloc median=4.0, non-altloc median=4.0; p=0.41).

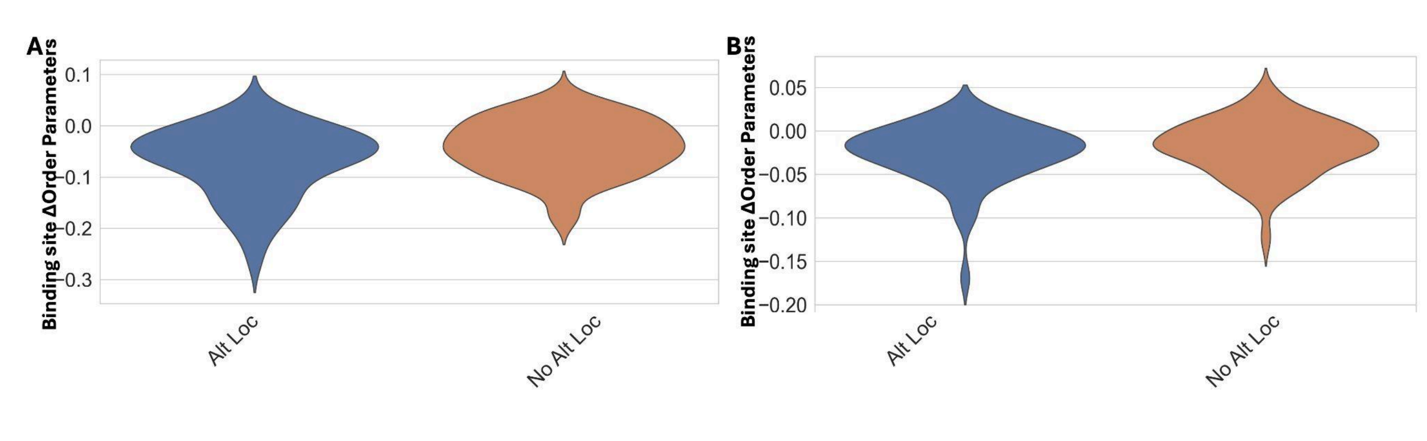

Supplementary Figure 10. A. Binding site order parameter difference (Ligands with modeled alt loc median:-0.059, Ligands without modeled alt loc median: -0.038; p=0.02), **B.** All protein order parameter differences (Ligands with modeled alt loc :-0.019, Ligands without modeled alt loc : -0.018; p=0.33).

bioRxiv preprint doi: https://doi.org/10.1101/2025.11.25.690589; this version posted December 23, 2025. The copyright holder for this preprint (which was not certified by peer review) is the author/funder, who has granted bioRxiv a license to display the preprint in perpetuity. It is made available under aCC-BY 4.0 International license.

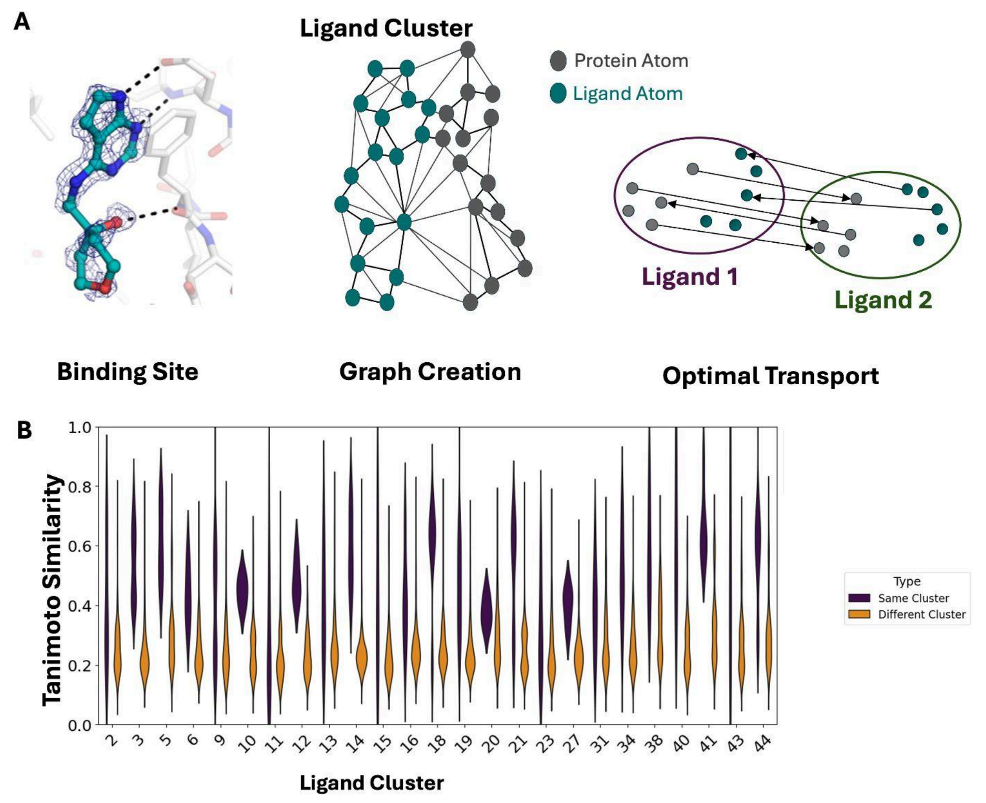

Supplementary Figure 11. **A**. Protein-ligand interaction optimal transport pipeline. **B.** Tanimoto similarity distribution among ligand clusters.

bioRxiv preprint doi: https://doi.org/10.1101/2025.11.25.690589; this version posted December 23, 2025. The copyright holder for this preprint (which was not certified by peer review) is the author/funder, who has granted bioRxiv a license to display the preprint in perpetuity. It is made available under aCC-BY 4.0 International license.

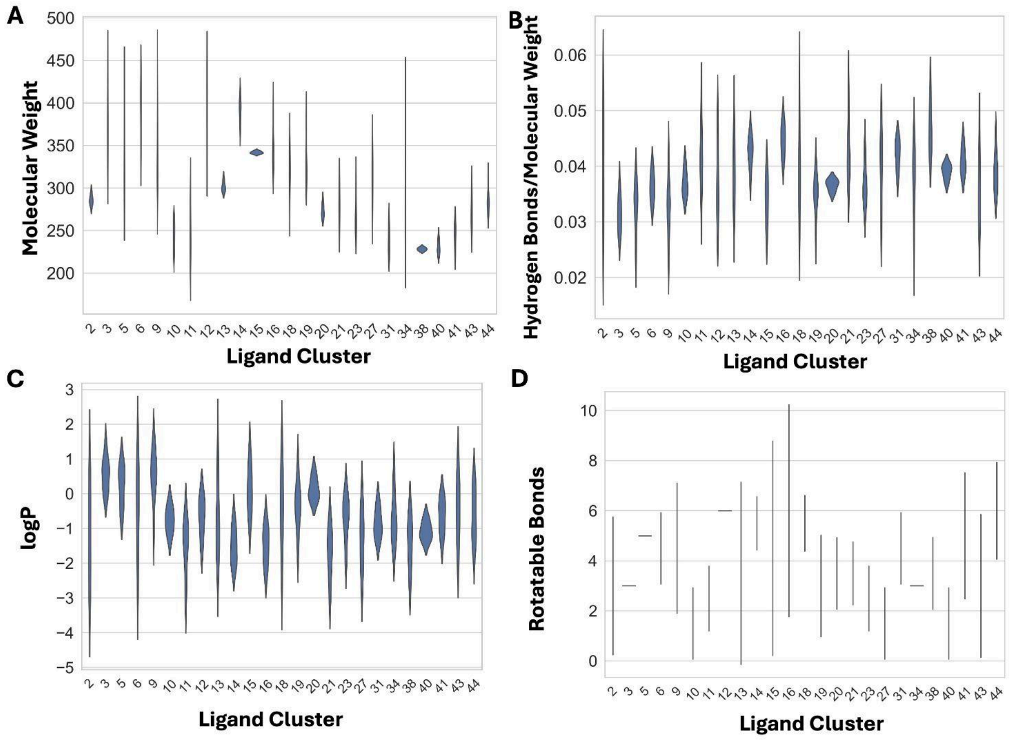

Supplementary Figure 12. Distribution of ligand properties among ligand clusters.

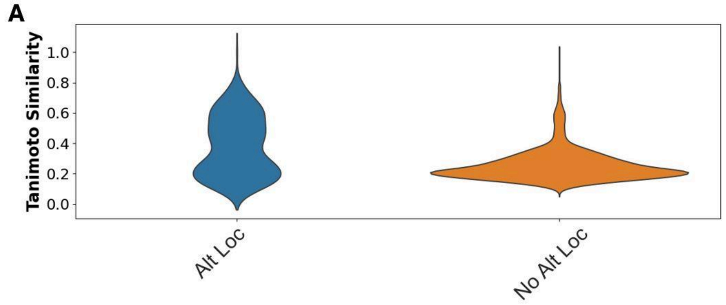

Supplementary Figure 13. Tanimoto similarity distribution among all ligand with alt locs (blue) vs. ligands without altlocs compared to all ligands with single conformers.

bioRxiv preprint doi: https://doi.org/10.1101/2025.11.25.690589; this version posted December 23, 2025. The copyright holder for this preprint (which was not certified by peer review) is the author/funder, who has granted bioRxiv a license to display the preprint in perpetuity. It is made available under aCC-BY 4.0 International license.

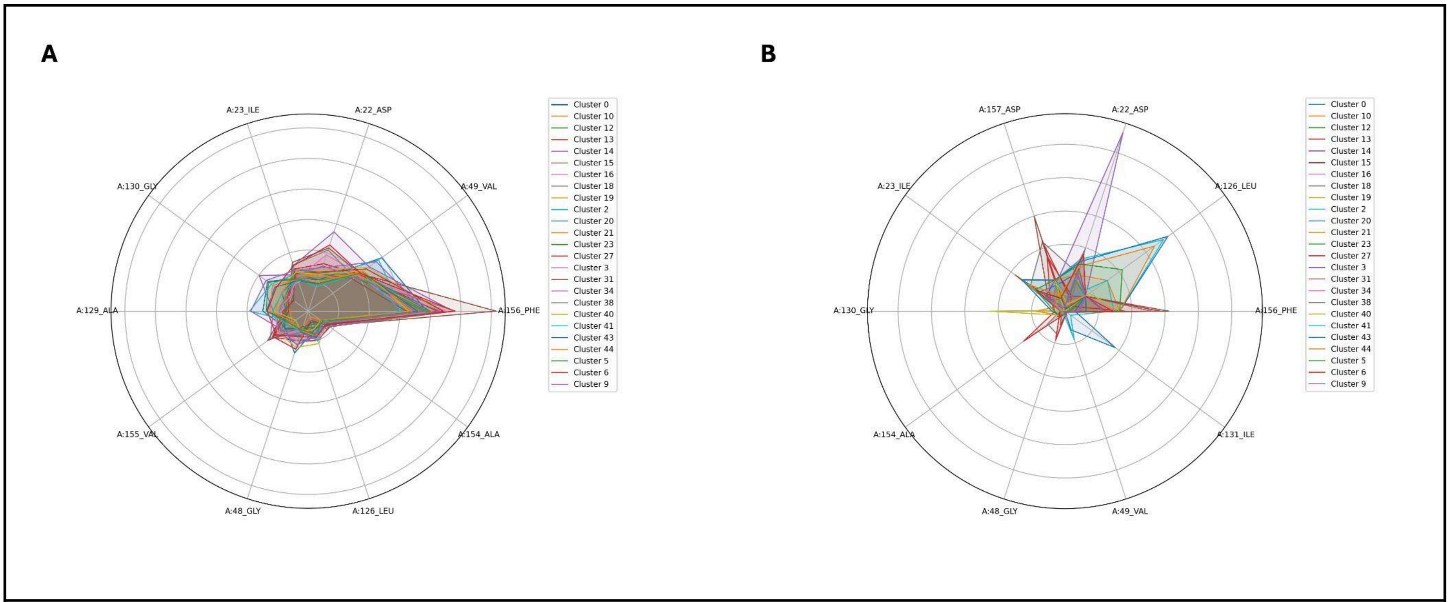

Supplementary Figure 14. Interaction frequency between binding site residues and ligand groups. **A.** All interactions, **B.** Hydrogen bonds interactions between binding site residues and ligand groups.

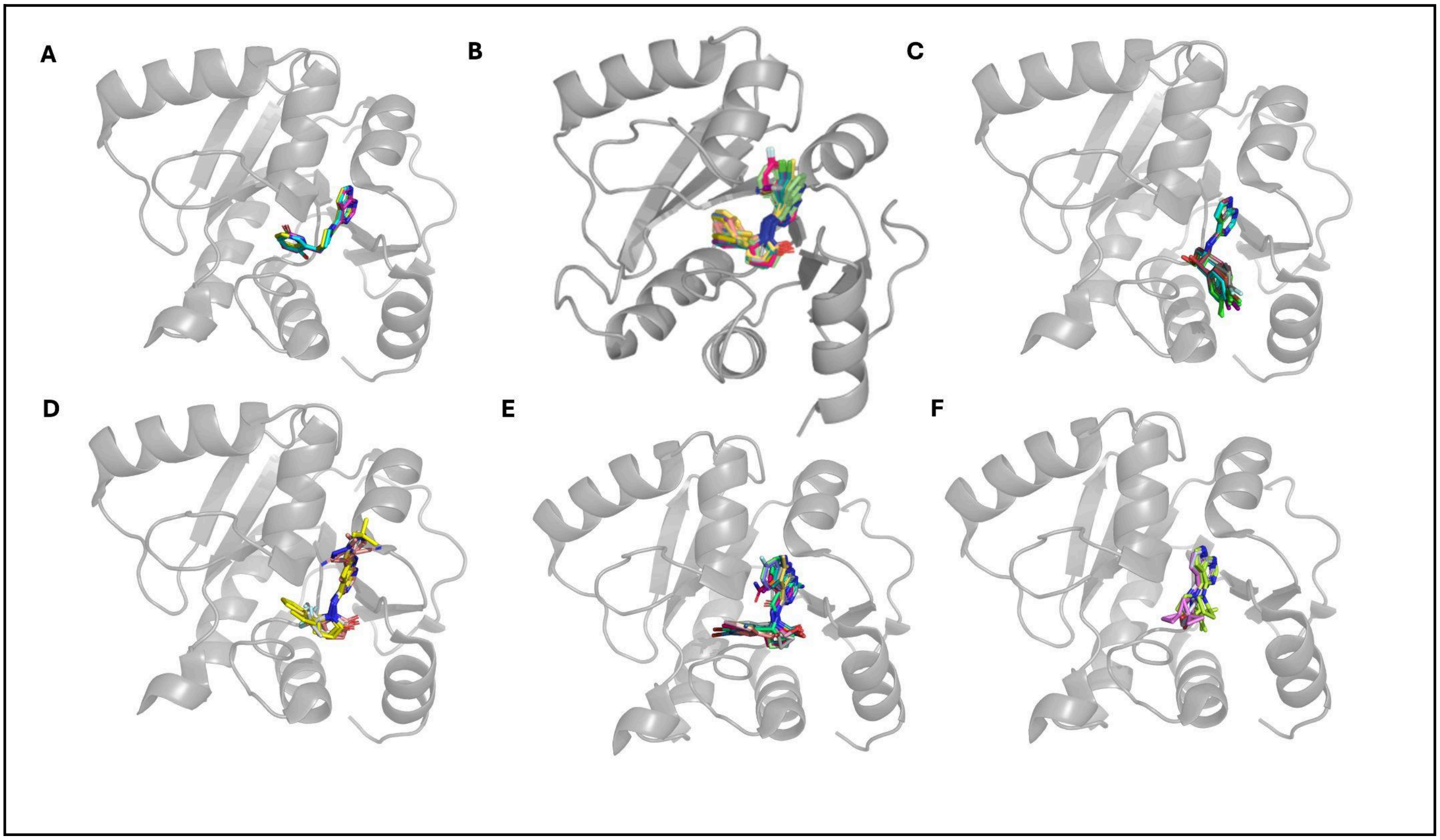

bioRxiv preprint doi: https://doi.org/10.1101/2025.11.25.690589; this version posted December 23, 2025. The copyright holder for this preprint (which was not certified by peer review) is the author/funder, who has granted bioRxiv a license to display the preprint in perpetuity. It is made available under aCC-BY 4.0 International license.

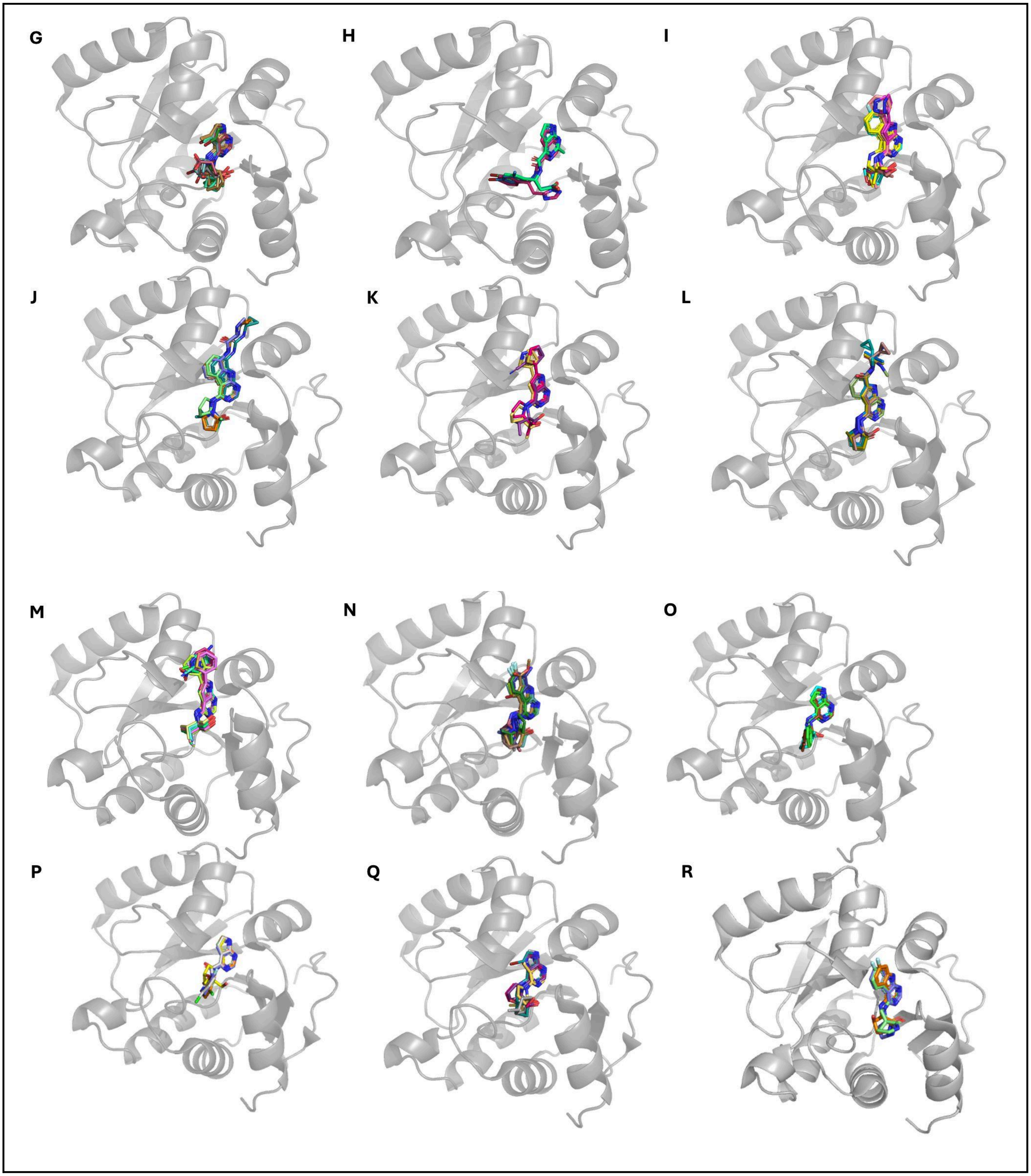

bioRxiv preprint doi: https://doi.org/10.1101/2025.11.25.690589; this version posted December 23, 2025. The copyright holder for this preprint (which was not certified by peer review) is the author/funder, who has granted bioRxiv a license to display the preprint in perpetuity. It is made available under aCC-BY 4.0 International license.

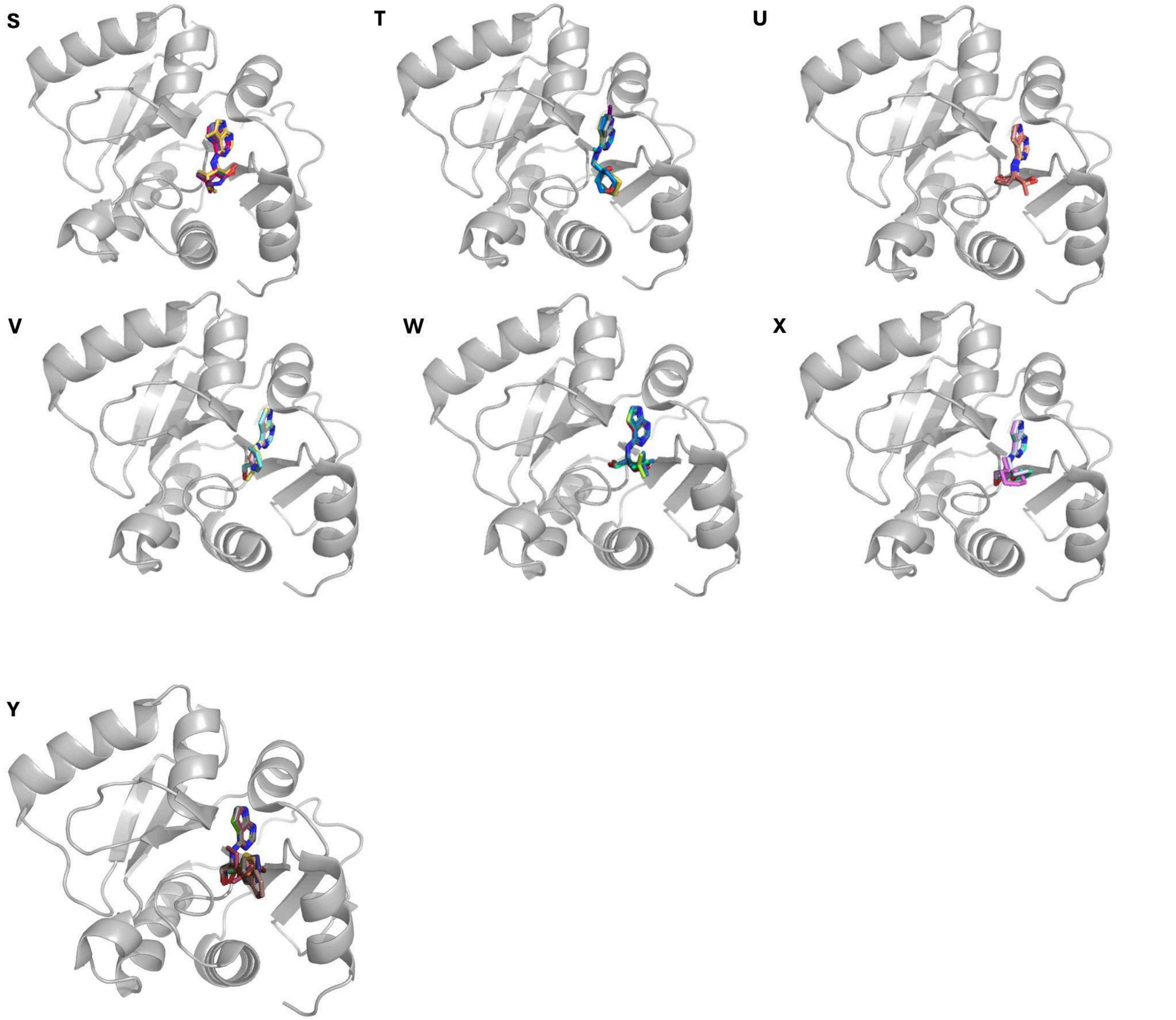

Supplementary Figure 15. A. Ligand Group 2, **B.** Ligand Group 3, **C.** Ligand Group 5, **D.** Ligand Group 6, **E.** Ligand Group 9, **F.** Ligand Group 10, **G.** Ligand Group 11, **H.** Ligand Group 12, **I.** Ligand Group 13, **J.** Ligand Group 14, **K.** Ligand Group 15, **L.** Ligand Group 16, **M.** Ligand Group 18, **N.** Ligand Group 19, **O.** Ligand Group 20, **P.** Ligand Group 21, **Q.** Ligand Group 23, **R.** Ligand Group 27, **S.** Ligand Group 31, **T.** Ligand Group 34, **U.** Ligand Group 38, **V.** Ligand Group 40, **W.** Ligand Group 41, **X.** Ligand Group 43, **Y.** Ligand Group 44

bioRxiv preprint doi: https://doi.org/10.1101/2025.11.25.690589; this version posted December 23, 2025. The copyright holder for this preprint (which was not certified by peer review) is the author/funder, who has granted bioRxiv a license to display the preprint in perpetuity. It is made available under aCC-BY 4.0 International license.

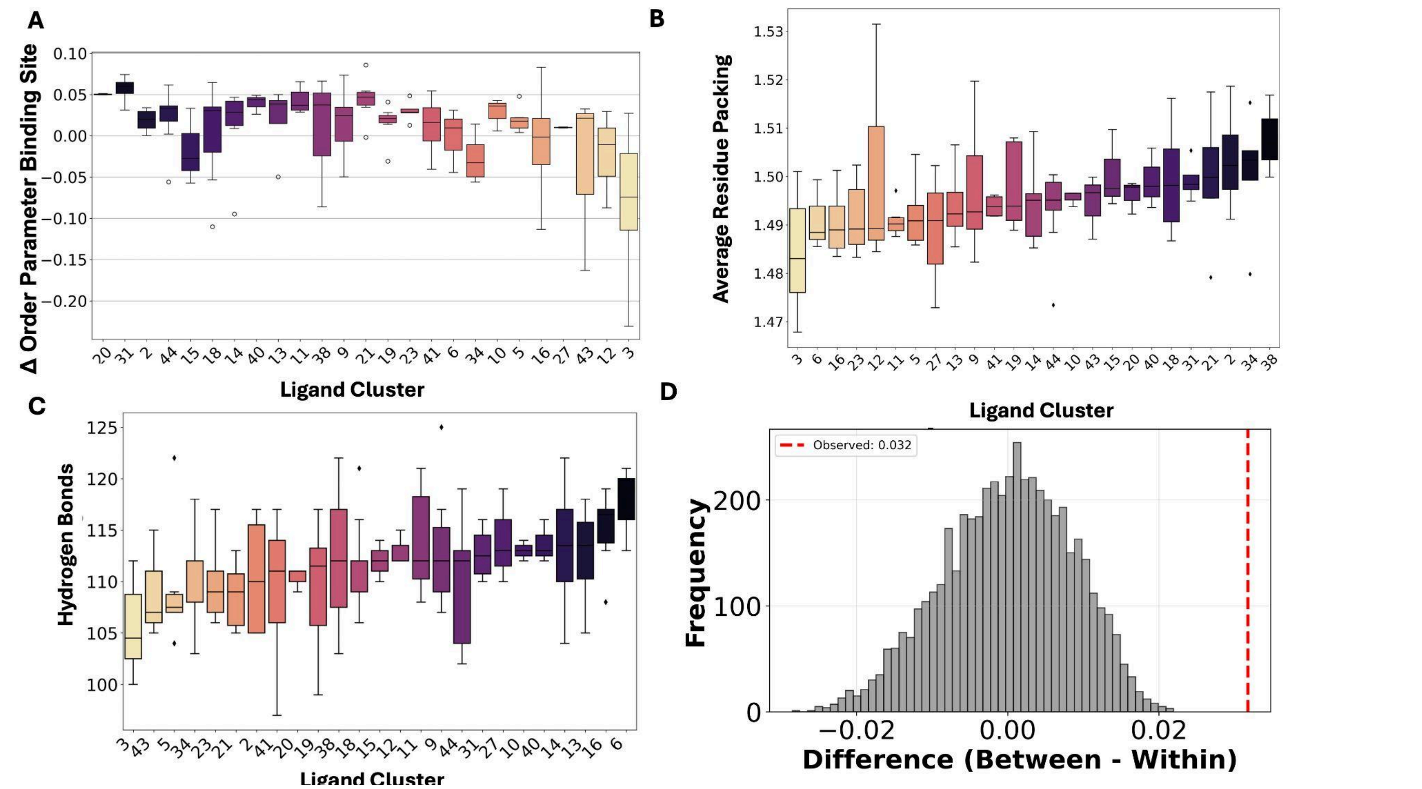

Supplementary Figure 16. A. ΔOrder Parameter in Binding Site per Ligand Group **B.** Packing by ligand group**. C.** Number of hydrogen bonds per ligand group. **D.** Permutation test results from 1-Pearson on order parameter differences.

bioRxiv preprint doi: https://doi.org/10.1101/2025.11.25.690589; this version posted December 23, 2025. The copyright holder for this preprint (which was not certified by peer review) is the author/funder, who has granted bioRxiv a license to display the preprint in perpetuity. It is made available under aCC-BY 4.0 International license.

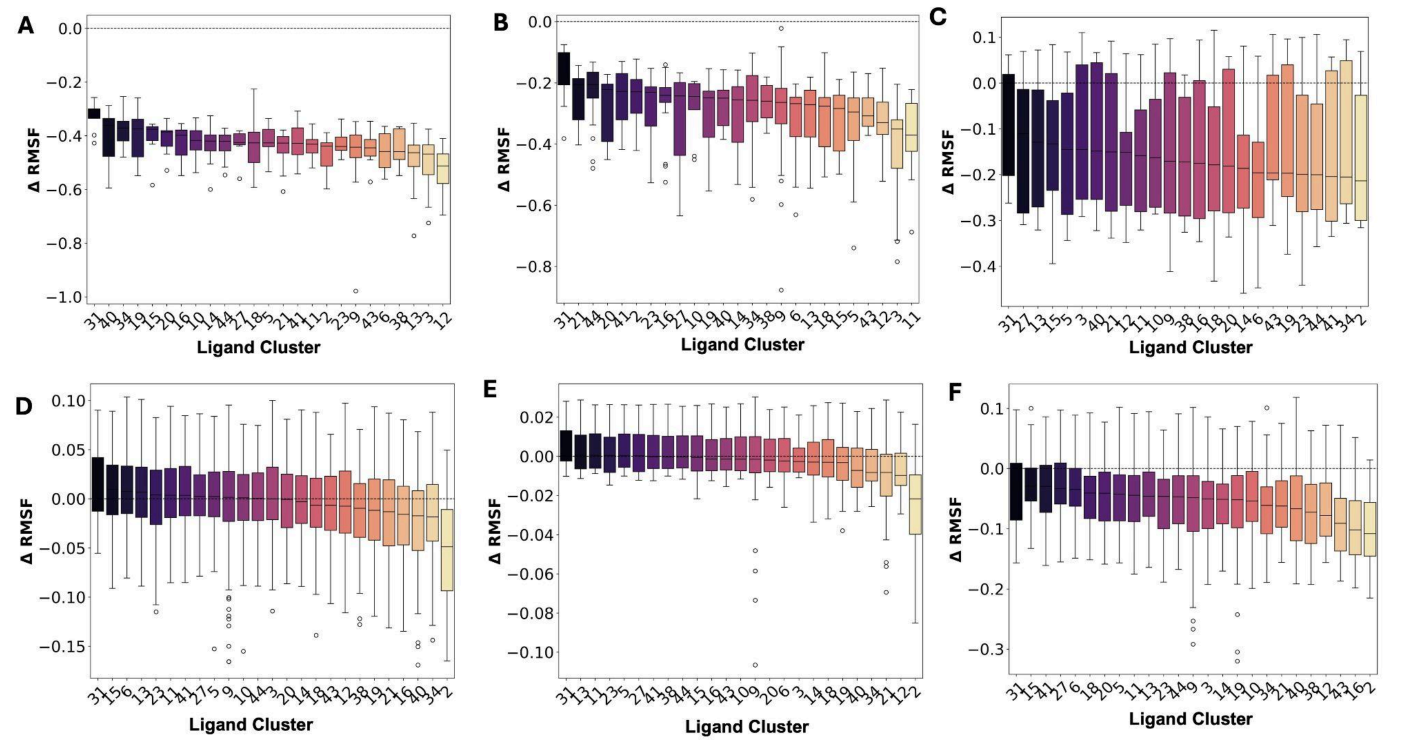

Supplementary Figure 17. RMSF for each residue cluster separated by ligand group. **A.** Residue Cluster 1 (residue number 102, 103, 104, 105, 106). **B.** Residue cluster 2 (residues 157, 158, 159, 161, 162, 165) **C.** Residue Cluster 3 (residues 29, 30, 31), **D.** Residue cluster 4 (residue numbers 36, 78, 79, 80, 81, 83, 93, 109, 110, 114, 115, 116, 117, 118, 119, 142, 143) **E.** Residue cluster 5 (37, 39, 45, 59, 60, 62, 94, 95), **F.** 13, 15, 16, 114, 146, 147, 149, 151, 152.

bioRxiv preprint doi: https://doi.org/10.1101/2025.11.25.690589; this version posted December 23, 2025. The copyright holder for this preprint (which was not certified by peer review) is the author/funder, who has granted bioRxiv a license to display the preprint in perpetuity. It is made available under aCC-BY 4.0 International license.

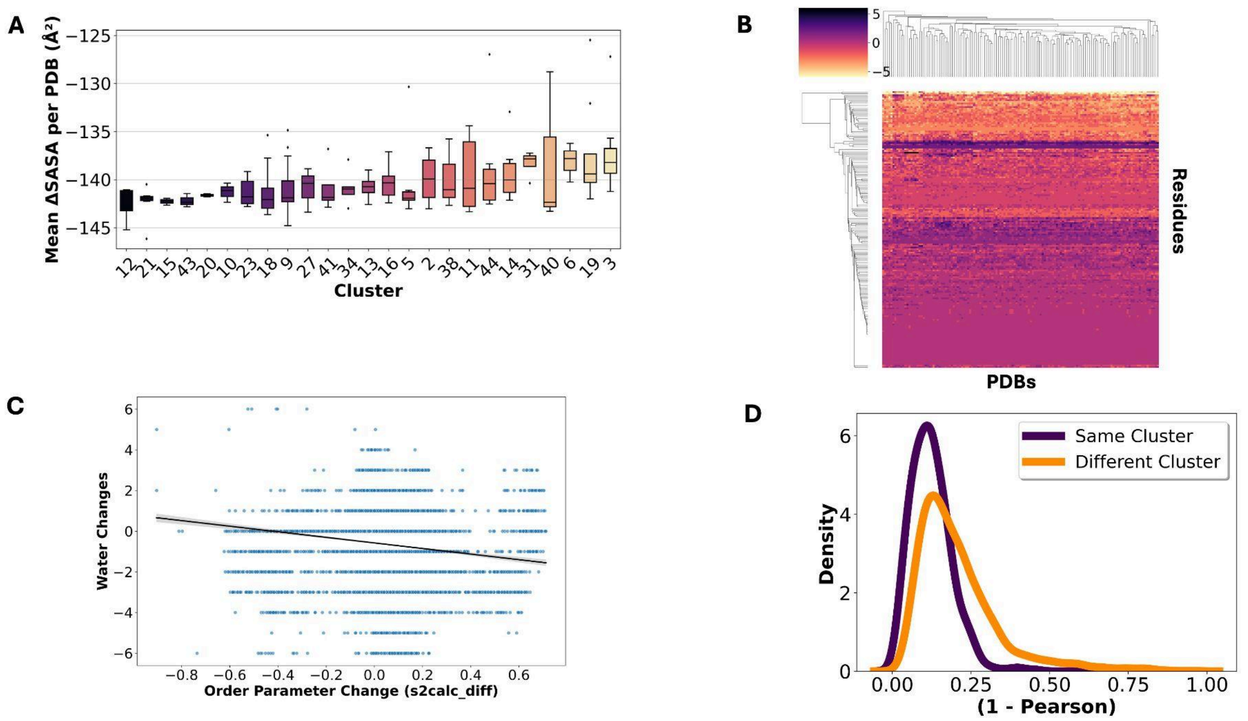

Supplementary Figure 18. A. Change in solvent accessible surface area by ligand group. Negative SASA indicates solvent accessible surface area lost upon ligand binding. **B.** Distribution of water molecules lost or gained across every PDB and every residue. **C.** KDE of water gain or loss exploring only binding site residues defined as any residues with 3 Å of any ligand heavy atom across the dataset (p=1.25e-55**;** Means: within=0.126, between=0.209).

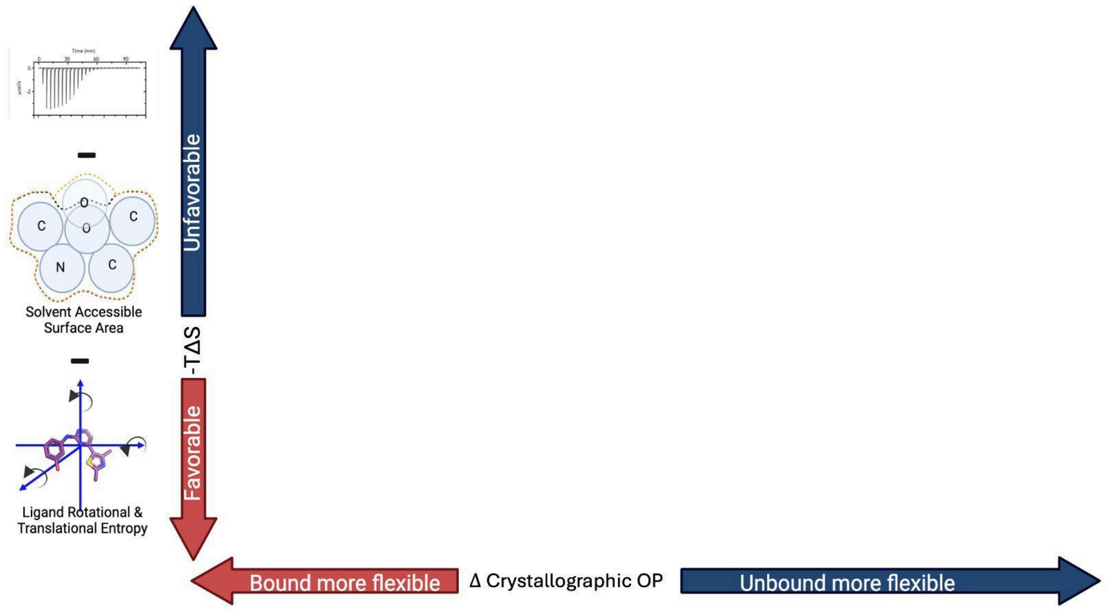

bioRxiv preprint doi: https://doi.org/10.1101/2025.11.25.690589; this version posted December 23, 2025. The copyright holder for this preprint (which was not certified by peer review) is the author/funder, who has granted bioRxiv a license to display the preprint in perpetuity. It is made available under aCC-BY 4.0 International license.

Supplementary Figure 19. Set up of ITC experiment. On the x-axis is the crystallographic order parameter estimate. On the y-axis is the ITC measured entropy, with solvent and ligand estimated entropy subtracted.

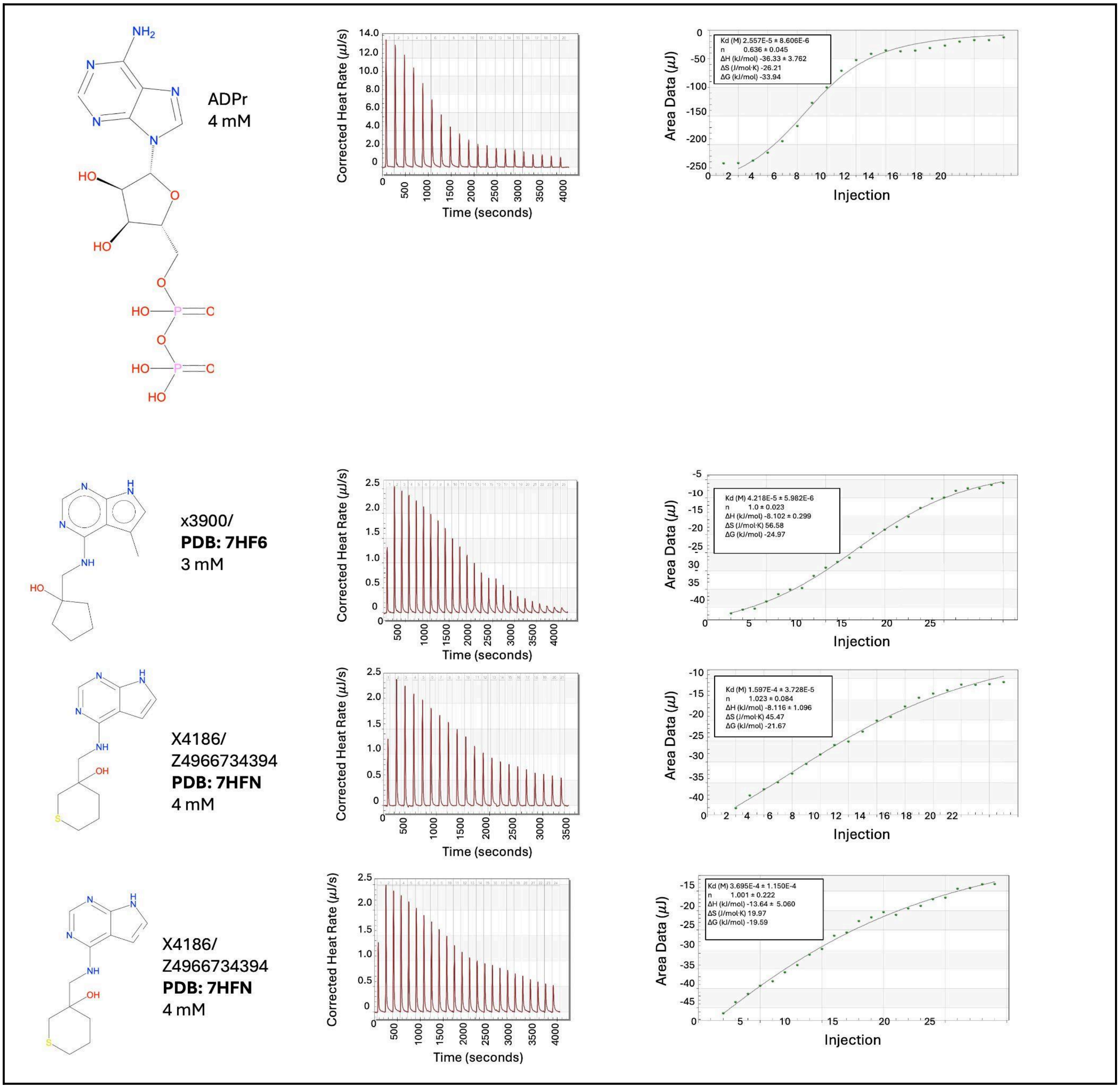

bioRxiv preprint doi: https://doi.org/10.1101/2025.11.25.690589; this version posted December 23, 2025. The copyright holder for this preprint (which was not certified by peer review) is the author/funder, who has granted bioRxiv a license to display the preprint in perpetuity. It is made available under aCC-BY 4.0 International license.

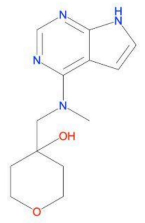

x4183/
Z1765463676/
PDB:7HFP
5 mM

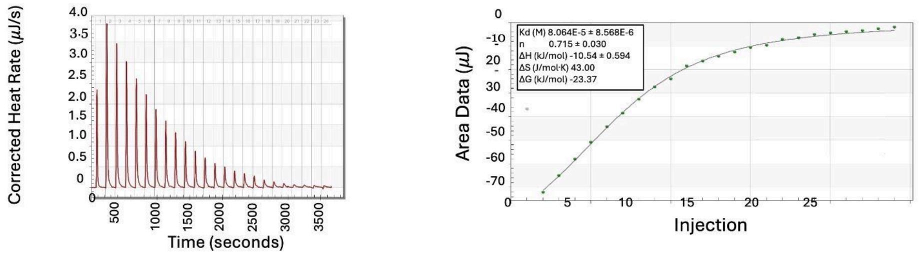

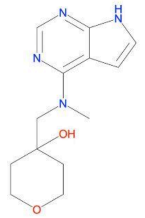

x4183/
Z1765463676/
PDB:7HFP
5 mM

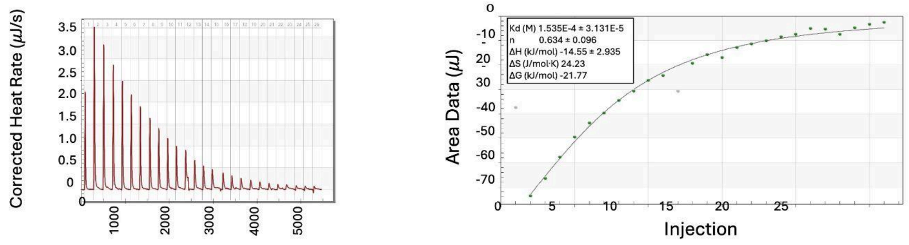

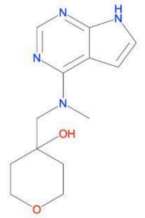

x4183/
Z1765463676/
PDB:7HFP
5 mM

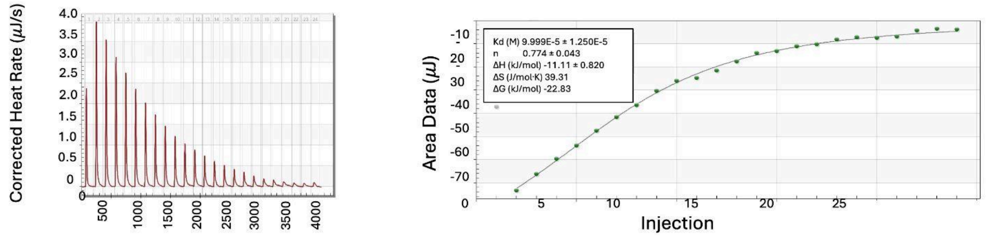

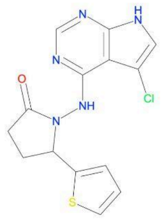

x3458/
Z77039868494
PDB: 13VC
4 mM

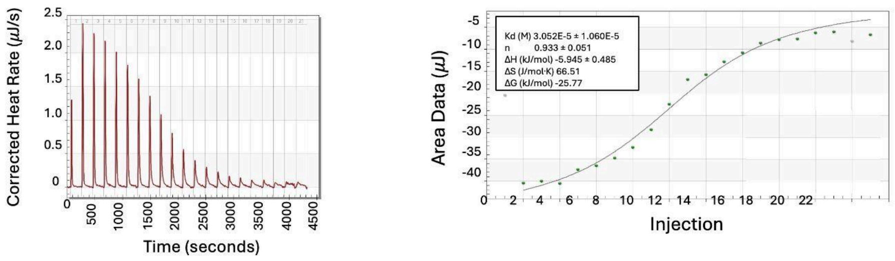

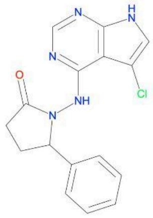

x3466/
Z7703986795
PDB: 7HI4
4 mM

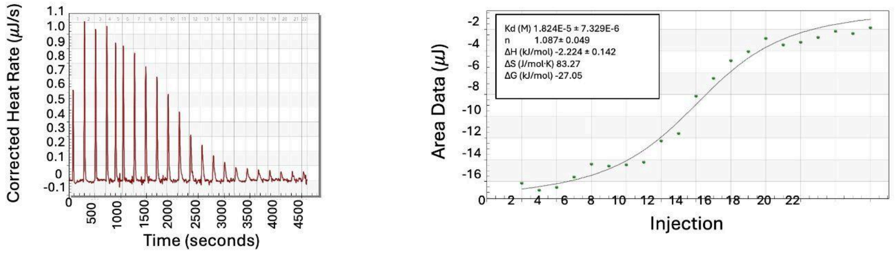

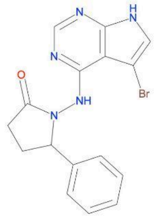

x3439/
Z7703984087
PDB: 7HHZ
3 mM

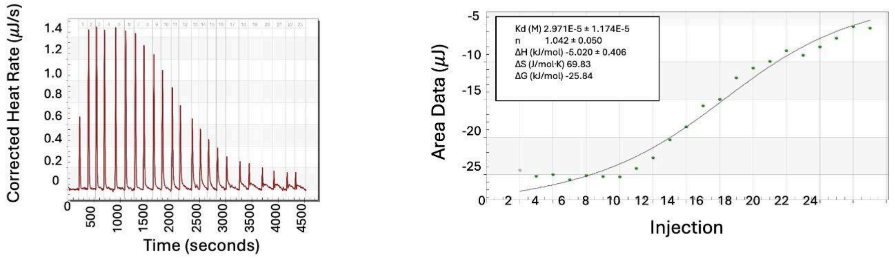

bioRxiv preprint doi: https://doi.org/10.1101/2025.11.25.690589; this version posted December 23, 2025. The copyright holder for this preprint (which was not certified by peer review) is the author/funder, who has granted bioRxiv a license to display the preprint in perpetuity. It is made available under aCC-BY 4.0 International license.

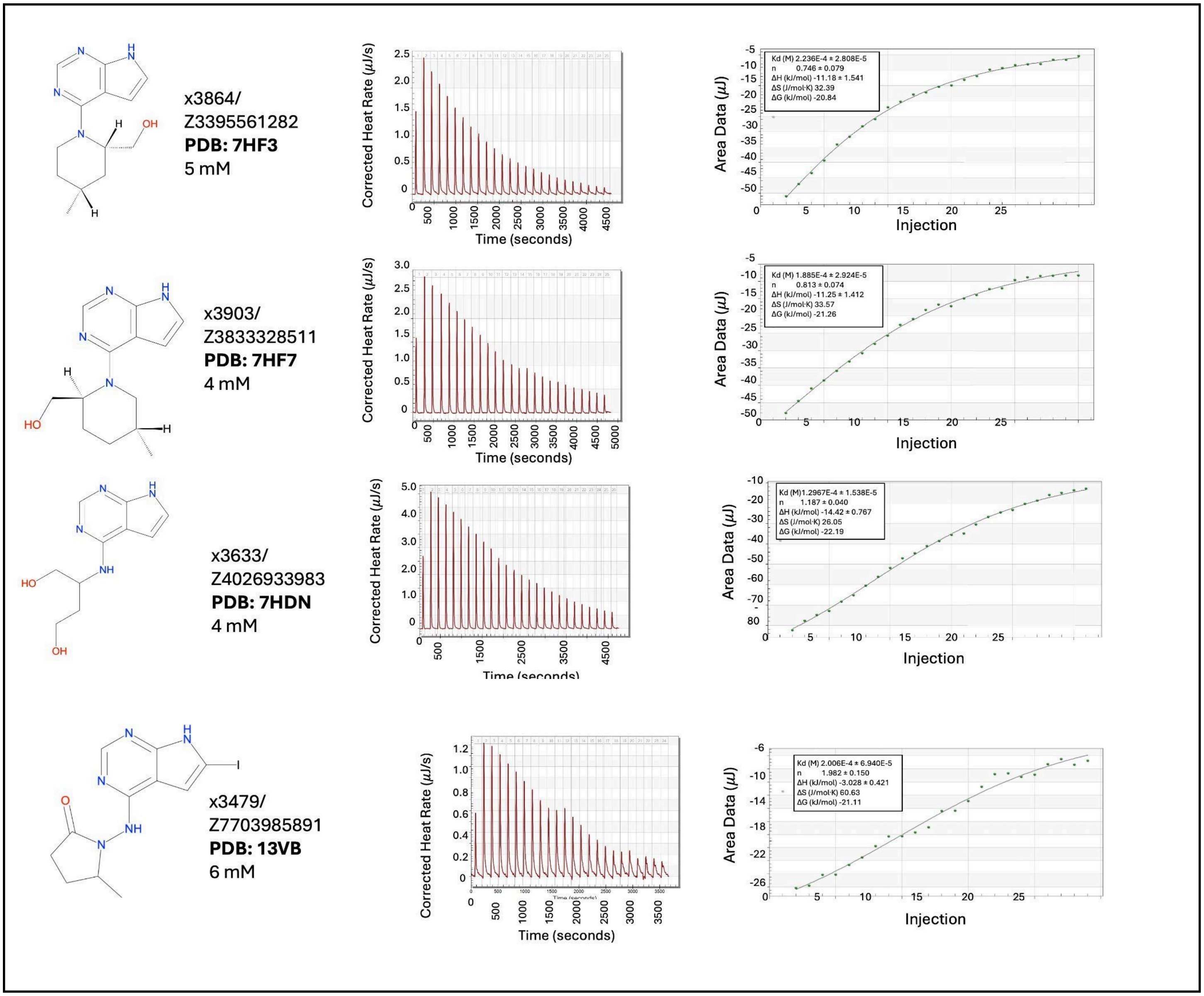

bioRxiv preprint doi: https://doi.org/10.1101/2025.11.25.690589; this version posted December 23, 2025. The copyright holder for this preprint (which was not certified by peer review) is the author/funder, who has granted bioRxiv a license to display the preprint in perpetuity. It is made available under aCC-BY 4.0 International license.

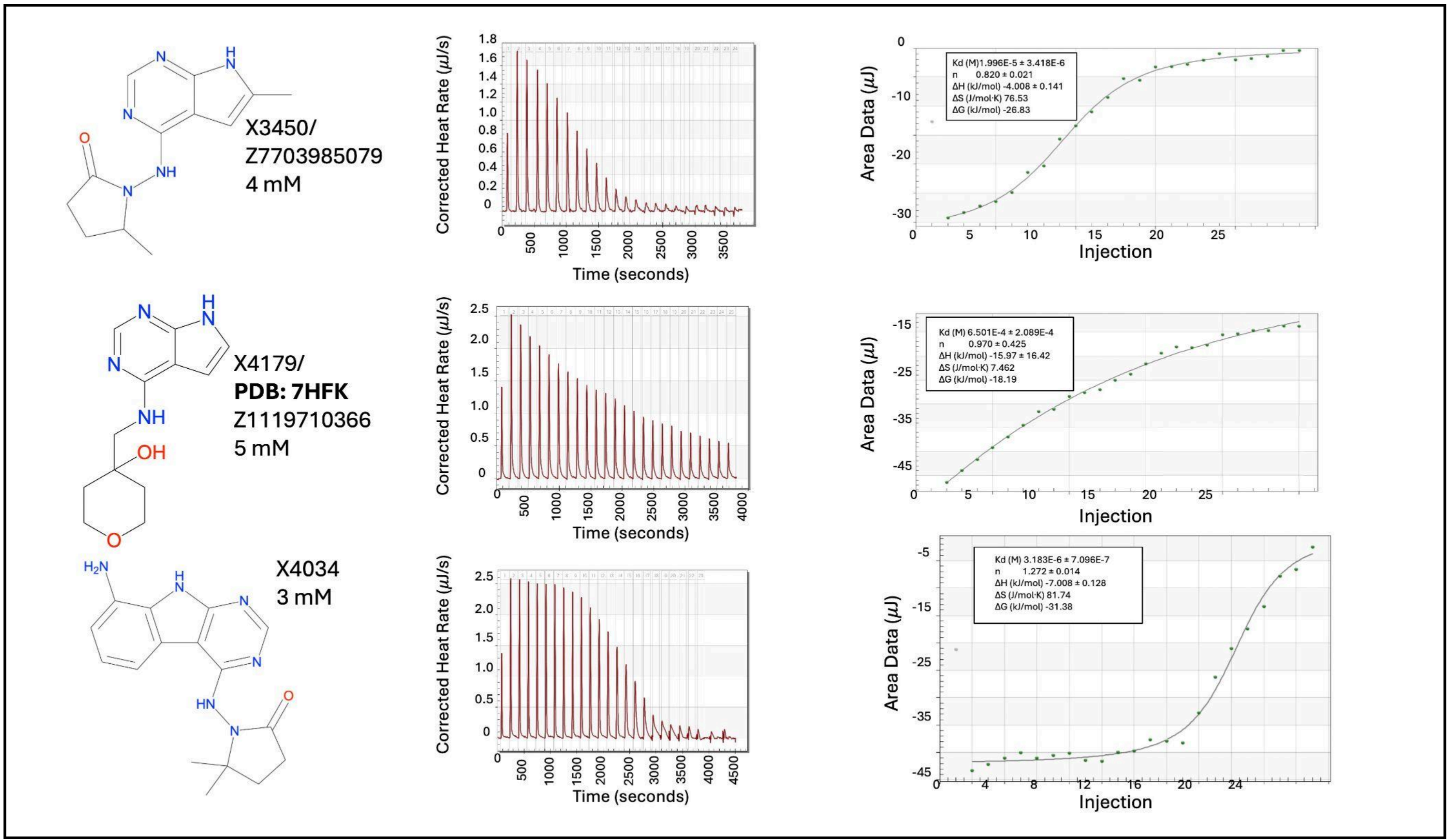

Supplementary Figure 20. Raw and calculated thermograms for all ligands. If ligands had multiple ITC measurements, we took the average of all runs. Ligands with Z values were ordered from enamine. Others were synthesized at UCSF with descriptions elsewhere.

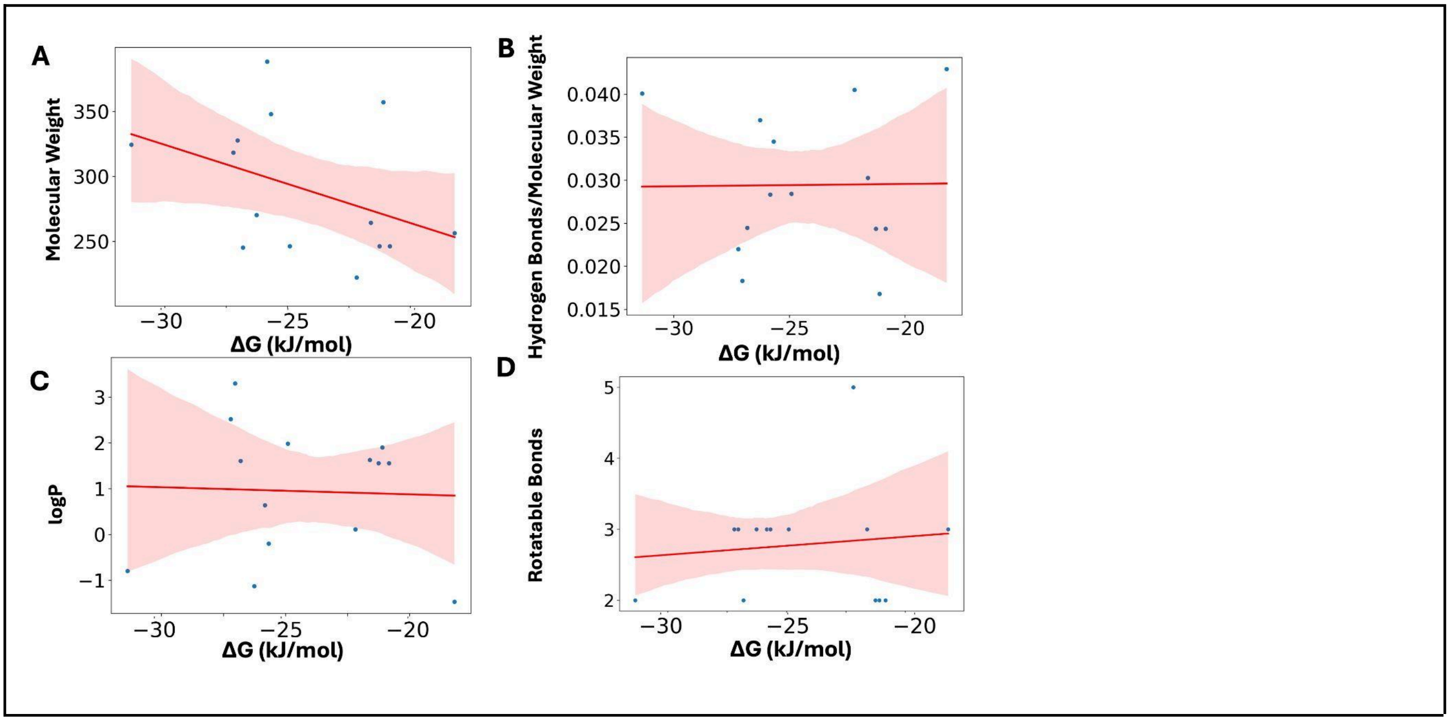

bioRxiv preprint doi: https://doi.org/10.1101/2025.11.25.690589; this version posted December 23, 2025. The copyright holder for this preprint (which was not certified by peer review) is the author/funder, who has granted bioRxiv a license to display the preprint in perpetuity. It is made available under aCC-BY 4.0 International license.

Supplementary Figure 21. Ligand Properties and Correlation with dG as measured with ITC. A. Molecular Weight (slope:-6.01; R^2:0.16; p=0.15) B. Hydrogen bonds per molecular weight (slope:0; R^2= 0.0001; p=0.97). C. LogP (slope:-0.02; R^2= 0.001; p=0.90) D. Rotatable bonds (slope:0.02; R^2= 0.01; p=0.71).

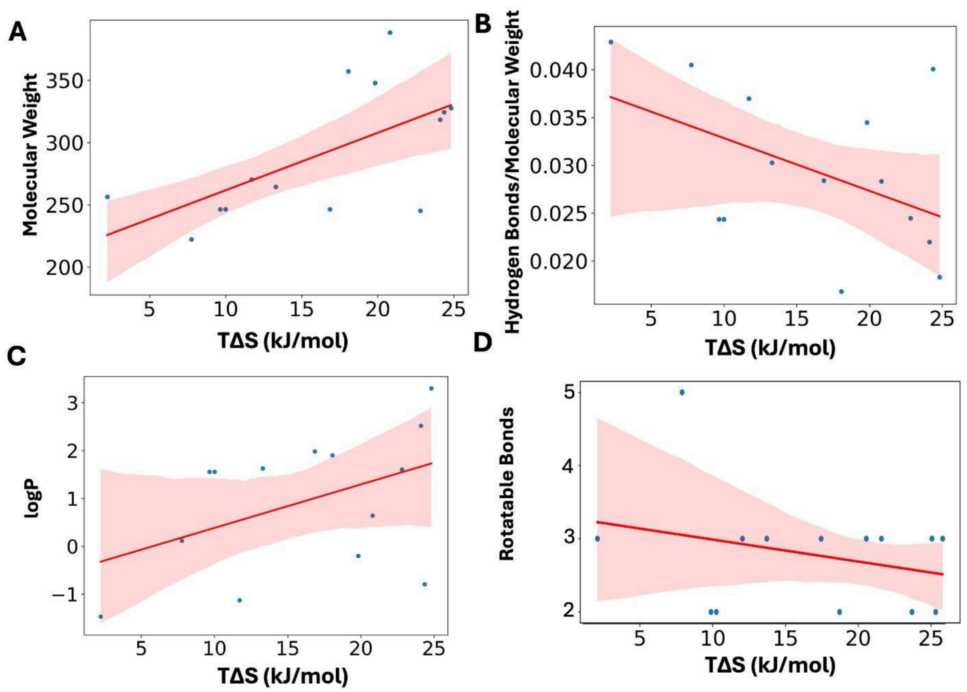

Supplementary Figure 22. Ligand Properties and Correlation with TΔS. A. Molecular Weight (slope:4.62; R^2= 0.40; p=0.015) B. Hydrogen bonds per molecular weight (slope:0.038 R^2= 0.22; p=0.091). C. LogP (slope=0.09; R^2= 0.20; p=0.11) D. Rotatable bonds (slope=-0.03; R^2= 0.08; p=0.33).
